# Early ventricular tachyarrhythmia after myocardial infarction in patients using a wearable cardioverter defibrillator

**DOI:** 10.1093/europace/euaf293

**Published:** 2025-11-12

**Authors:** Johannes Rips, Ibrahim El-Battrawy, Mustafa Mousa Basha, Hendrik Lapp, Andreas Zietzer, Vincent Knappe, Maximilian Funken, Christopher Gestrich, Tobias Kratz, Leonie Wloch, Katharina Koepsel, Norbert Klein, Thomas Kuntz, Andreas Mügge, Assem Aweimer, Christian Weth, Florian Custodis, Wasim Schehab, Luisa Marie Hoffmann, Mohammad Abumayyaleh, Ibrahim Akin, Nazha Hamdani, Georg Nickenig, Thomas Beiert

**Affiliations:** Heart Centre Bonn, Department of Medicine II, University Hospital Bonn, Venusberg-Campus 1, Bonn D-53127, Germany; Department of Cardiology and Rhythmology, St. Josef Hospital, Ruhr University Bochum, Bochum, Germany; Department of Molecular and Experimental Cardiology, Institut für Forschung und Lehre (IFL), Ruhr University Bochum, Bochum, Germany; Heart Centre Bonn, Department of Medicine II, University Hospital Bonn, Venusberg-Campus 1, Bonn D-53127, Germany; Heart Centre Bonn, Department of Medicine II, University Hospital Bonn, Venusberg-Campus 1, Bonn D-53127, Germany; Heart Centre Bonn, Department of Medicine II, University Hospital Bonn, Venusberg-Campus 1, Bonn D-53127, Germany; Heart Centre Bonn, Department of Medicine II, University Hospital Bonn, Venusberg-Campus 1, Bonn D-53127, Germany; Heart Centre Bonn, Department of Medicine II, University Hospital Bonn, Venusberg-Campus 1, Bonn D-53127, Germany; Heart Centre Bonn, Department of Medicine II, University Hospital Bonn, Venusberg-Campus 1, Bonn D-53127, Germany; Department of Paediatric Cardiology, University Hospital Bonn, Bonn, Germany; Department of Cardiology and Rhythmology, St. Josef Hospital, Ruhr University Bochum, Bochum, Germany; Department of Molecular and Experimental Cardiology, Institut für Forschung und Lehre (IFL), Ruhr University Bochum, Bochum, Germany; Department of Cardiology and Rhythmology, St. Josef Hospital, Ruhr University Bochum, Bochum, Germany; Department of Cardiology, Angiology and Internal Intensive-Care Medicine, Klinikum St. Georg gGmbH Leipzig, Leipzig, Germany; Department of Cardiology, Angiology and Internal Intensive-Care Medicine, Klinikum St. Georg gGmbH Leipzig, Leipzig, Germany; Department of Cardiology and Rhythmology, St. Josef Hospital, Ruhr University Bochum, Bochum, Germany; Department of Molecular and Experimental Cardiology, Institut für Forschung und Lehre (IFL), Ruhr University Bochum, Bochum, Germany; Department of Cardiology and Angiology, Clinic Saarbrücken gGmbH, Saarbrücken, Germany; Department of Cardiology and Angiology, Clinic Saarbrücken gGmbH, Saarbrücken, Germany; Department of Internal Medicine II Cardiology, Angiology, Ludmillenstift Hospital, Meppen, Germany; Department of Cardiology, Hemostaseology and Medical Intensive Care, University Medical Centre Mannheim, Medical Faculty Mannheim, Heidelberg University, Mannheim, Germany; Department of Cardiology, Hemostaseology and Medical Intensive Care, University Medical Centre Mannheim, Medical Faculty Mannheim, Heidelberg University, Mannheim, Germany; Department of Cardiology, Hemostaseology and Medical Intensive Care, University Medical Centre Mannheim, Medical Faculty Mannheim, Heidelberg University, Mannheim, Germany; Department of Cardiology and Rhythmology, St. Josef Hospital, Ruhr University Bochum, Bochum, Germany; Department of Molecular and Experimental Cardiology, Institut für Forschung und Lehre (IFL), Ruhr University Bochum, Bochum, Germany; Heart Centre Bonn, Department of Medicine II, University Hospital Bonn, Venusberg-Campus 1, Bonn D-53127, Germany; Heart Centre Bonn, Department of Medicine II, University Hospital Bonn, Venusberg-Campus 1, Bonn D-53127, Germany

**Keywords:** Wearable cardioverter defibrillator, Myocardial infarction, Ischaemic cardiomyopathy, Ventricular tachyarrhythmia, Implantable cardioverter defibrillator

## Introduction

Patients with a severely reduced left ventricular ejection fraction (LV-EF) due to ischaemic cardiomyopathy (ICM) are at increased risk of sudden cardiac death (SCD).^1^ The current ESC guidelines therefore recommend implantable cardioverter defibrillator (ICD) therapy in patients with a LV-EF ≤ 35% after at least three months of optimized medical therapy (OMT), but refrain from ICD implantation in the early phase after myocardial infarction (MI).^2–4^ The wearable cardioverter defibrillator (WCD) might serve as an alternative to protect patients with a newly diagnosed severely reduced LV-EF in the early phase after MI without the downsides of implantable electronic devices. The aim of the present study was to determine whether WCD therapies or ventricular tachyarrhythmia differ between patients with high risk for SCD following ST-elevation MI (STEMI) or non-ST-elevation MI (NSTEMI).

## Methods

This multicentre study included patients receiving a WCD for increased risk for SCD (LV-EF ≤ 35% and/or non-sustained ventricular tachycardia) following an acute MI. Follow-up was performed at 3 months.

Normally distributed continuous variables are shown as mean ± standard deviation. Categorical variables are expressed as counts with percentages. Comparisons, using Fisher’s exact tests for categorial variables and independent *t*-tests for continuous variables, were performed as appropriate. LV-EF changes over time were assessed with paired *t*-tests. Time-to-event outcomes are shown as Kaplan–Meier curves and compared with the log-rank test. Individual risk factors were evaluated using Cox regression analysis. Two-sided *P*-values < 0.05 were considered statistically significant.

## Results

### Baseline characteristics

A total of 272 patients (STEMI: 118; NSTEMI: 154; 86.4% male) were included. STEMI patients were younger (STEMI: 61.9 ± 12.8 years vs. NSTEMI: 67.6 ± 11.5 years; *P* < 0.001), and more frequently active smokers (50.8% vs. 33.8%; *P* = 0.047). Prior MI was more common in STEMI patients (73.3% vs. 60.4%; *P* = 0.021), whereas prior coronary artery bypass grafting was more common in NSTEMI patients (5.9% vs. 20.1%; *P* < 0.001). There were more women in the NSTEMI group (8.5% vs. 17.5%; *P* = 0.033). Electrocardiogram parameters showed a longer QRS duration in NSTEMI patients (106.4 ± 26.8 ms vs. 115.3 ± 28.5 ms; *P* = 0.027). Regarding medical therapy at discharge, aspirin was prescribed more often in STEMI patients (91.5% vs. 79.4%; *P* = 0.011). OMT was initiated with no significant differences between groups. Mean daily wear time of the WCD did not differ significantly between groups (21.9 ± 3.5 h).

### Ejection fraction

**Figure 1:**
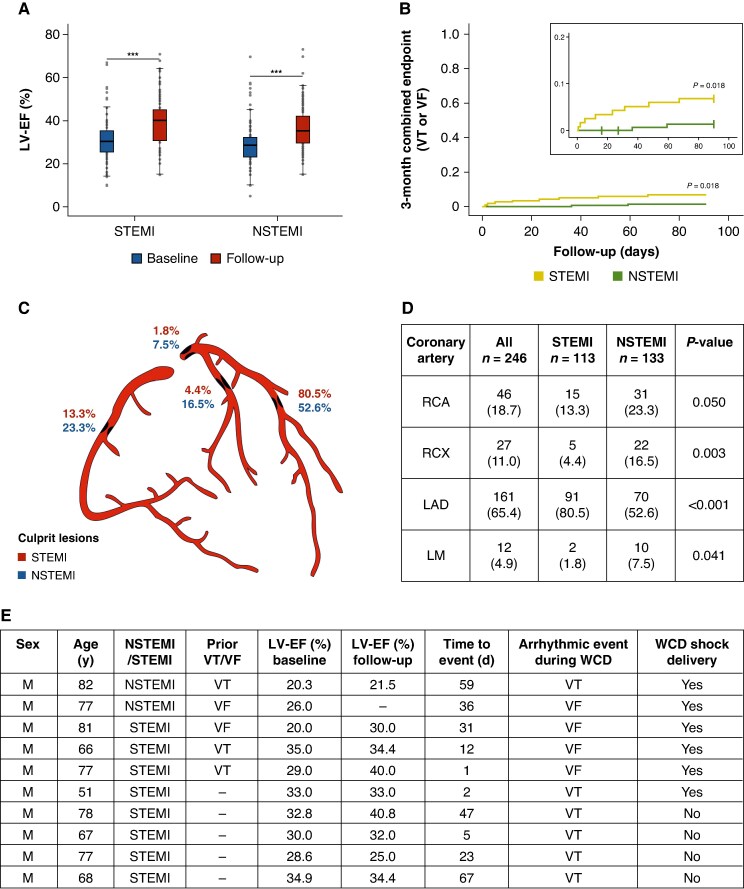
(*A***)** comparing LV-EF at baseline and 3-month follow-up between STEMI and NSTEMI. Boxplots display the distribution of LV-EF at baseline and at 3-month follow-up in both groups. Boxes represent the interquartile range (IQR) and horizontal lines indicate the median. (*B***)** Kaplan–Meier survival analysis of the primary endpoint (VT/VF) at three months, stratified by STEMI and NSTEMI. (*C* and *D***)** Distribution of culprit lesions depending on the type of MI. (*E***)** Detailed characteristics of patients with sustained VT/VF events during WCD use. F indicates female sex/M indicates male sex; LAD, left anterior descending artery; LM, left main artery; LV-EF, left ventricular ejection fraction; NSTEMI, Non-ST-elevation myocardial infarction; RCA, right coronary artery; RCX, circumflex artery; STEMI, ST-elevation myocardial infarction; VT/VF, ventricular tachycardia/ventricular fibrillation; WCD, wearable cardioverter defibrillator.

Although baseline LV-EF was slightly higher in the STEMI group (31.0 ± 9.1% vs. 28.7 ± 9.5%; *P* = 0.042; *Figure 1A*), both groups experienced a comparable increase in LV-EF (8.5 ± 10.6% vs. 7.8 ± 10.8%; *P* = 0.609). LV-EF increased to 39.2 ± 11.2% at follow-up in STEMI patients (*P* < 0.001) and to 36.1 ± 10.7% in the NSTEMI group (*P* < 0.001).

### Clinical outcomes

Patients with STEMI experienced significantly more ventricular tachycardia/ventricular fibrillation (VT/VF) episodes compared with NSTEMI patients (6.8% vs. 1.3%; *P* = 0.023). In total, 17 VT/VF events were recorded during follow-up, with 15 events occurring in the STEMI group (9 sustained VTs, 6 VF events) and only 2 events in the NSTEMI group (1 VT, 1 VF). An appropriate WCD shock was delivered in six patients (2.2%) with no significant differences between groups (*P* = 0.408). A detailed list of events during WCD-use is shown in *Figure 1E*. Kaplan–Meier analysis demonstrated a statistically significant difference in time to first VT/VF event between STEMI and NSTEMI patients (log-rank test, *P* = 0.018; *Figure 1B*).

In a multivariate analysis of the complete cohort, STEMI [hazard ratio (HR) = 4.05; *P* = 0.038] and chronic kidney disease (CKD) (HR = 6.21; *P* = 0.021) were independent predictors for VT/VF events.

### Culprit lesions

In 80.5% of STEMI patients the left anterior descending artery (LAD) was judged as culprit compared with only 52.6% in NSTEMI patients (*P* < 0.001) (*Figure* *1C* and *D*). Further differences were observed regarding left main artery (1.8% vs. 7.5%; *P* = 0.041) and circumflex artery (RCX) (4.4% vs. 16.5%; *P* = 0.003).

## Discussion

The main findings of our study can be summarized as followed:

1. STEMI patients had a significantly higher incidence of VT/VF events during the first three months compared with NSTEMI patients.
2. Both groups received OMT for heart failure and showed a similar significant improvement in LV-EF, although baseline and follow-up LV-EF remained lower in the NSTEMI group.
3. STEMI and CKD were identified as independent predictors of VT/VF events.

Despite the NSTEMI cohort being older and having a lower LV-EF and both groups being on OMT, STEMI patients suffered from a higher arrhythmic risk. The STEMI group showed a significantly higher prevalence of culprit lesions in the LAD, which are known to be more often associated with ventricular tachyarrhythmia than culprit lesions in other coronary vessels.^5,6^ Taken together, this study highlights the need for a more complex risk stratification and a close surveillance after hospital discharge, especially in STEMI patients. The WCD may pose a relevant role in this approach.

The VEST trial showed no significant reduction in arrhythmic mortality after MI by using the WCD.^7^ But regarding the low compliance as well as the per-protocol analysis showing a reduction in total and arrhythmic mortality the evidence is unclear.^8^ Moreover, the older DINAMIT and IRIS trials showed no improvement in overall mortality following ICD implantation early after MI.^9,10^ Nevertheless, the management of patients with severely reduced LV-EF has evolved considerably since the publication of those landmark studies with SCD prevention now favouring a patient-specific approach rather than one based solely on LV-EF which is in line with our results.

In the IRIS trial, patients were enrolled up to 13 days after MI without improvement in overall mortality following ICD implantation.^10^ In our study, three STEMI patients had a VT/VF event within this period, and half of patients experienced their first ventricular arrhythmia within 30 days post MI. Therefore, this study should be considered in the context of a reassessment of the ESC recommendation regarding the use of WCD in otherwise unprotected ICM patients early after STEMI, which is currently limited to a Class IIb.^2,3^

## Limitations

Data were collected and analysed retrospectively, and the study spans many years with multiple advances in heart failure therapy.

## Data Availability

Data supporting the study are curated at the Heart Center Bonn, University Hospital Bonn, Germany. These data are not shared openly but are available on reasonable request from the corresponding authors.

## References

[1] Moss AJ, Zareba W, Hall WJ, Klein H, Wilber DJ, Cannom DS et al Prophylactic implantation of a defibrillator in patients with myocardial infarction and reduced ejection fraction. N Engl J Med 2002;346:877–83. 11907286 10.1056/NEJMoa013474

[2] McDonagh TA, Metra M, Adamo M, Gardner RS, Baumbach A, Böhm M et al 2021 ESC guidelines for the diagnosis and treatment of acute and chronic heart failure. Eur Heart J 2021;42:3599–726. 34447992 10.1093/eurheartj/ehab368

[3] Zeppenfeld K, Tfelt-Hansen J, de Riva M, Winkel BG, Behr ER, Blom NA et al 2022 ESC guidelines for the management of patients with ventricular arrhythmias and the prevention of sudden cardiac death. Eur Heart J 2022;43:3997–4126. 36017572 10.1093/eurheartj/ehac262

[4] Könemann H, Dagres N, Merino JL, Sticherling C, Zeppenfeld K, Tfelt-Hansen J et al Spotlight on the 2022 ESC guideline management of ventricular arrhythmias and prevention of sudden cardiac death: 10 novel key aspects. Europace 2023;25:euad091. 37102266 10.1093/europace/euad091 PMC10228619

[5] Warming PE, Glinge C, Dusi V, Jabbari R, Stampe NK, Tan HL et al Risk factors associated with ventricular fibrillation during first ST-elevation myocardial infarction: individual participant data analysis of 3 prospective case-control studies. Heart Rhythm 2025;2:1547–5271. 10.1016/j.hrthm.2025.06.026 40778905

[6] Wasmer K, Reinecke H, Heitmann M, Dechering DG, Reinke F, Lange PS et al Clinical, procedural and long-term outcome of ischemic VT ablation in patients with previous anterior versus inferior myocardial infarction. Clin Res Cardiol 2020;109:1282–91. 32157380 10.1007/s00392-020-01622-z PMC7515937

[7] Olgin JE, Pletcher MJ, Vittinghoff E, Wranicz J, Malik R, Morin DP et al Wearable cardioverter–defibrillator after myocardial infarction. N Engl J Med 2018;379:1205–15. 30280654 10.1056/NEJMoa1800781 PMC6276371

[8] Olgin JE, Lee BK, Vittinghoff E, Morin DP, Zweibel S, Rashba E et al Impact of wearable cardioverter-defibrillator compliance on outcomes in the VEST trial: as-treated and per-protocol analyses. J Cardiovasc Electrophysiol 2020;31:1009–18. 32083365 10.1111/jce.14404 PMC9374026

[9] Hohnloser SH, Kuck KH, Dorian P, Roberts RS, Hampton JR, Hatala R et al Prophylactic use of an implantable cardioverter–defibrillator after acute myocardial infarction. N Engl J Med 2004;351:2481–8. 15590950 10.1056/NEJMoa041489

[10] Steinbeck G, Andresen D, Seidl K, Brachmann J, Hoffmann E, Wojciechowski D et al Defibrillator implantation early after myocardial infarction. N Engl J Med 2009;361:1427–36. 19812399 10.1056/NEJMoa0901889

